# Neighborhood fast food restaurants and fast food consumption: A national study

**DOI:** 10.1186/1471-2458-11-543

**Published:** 2011-07-08

**Authors:** Andrea S Richardson, Janne Boone-Heinonen, Barry M Popkin, Penny Gordon-Larsen

**Affiliations:** 1 Department of Nutrition, University of North Carolina at Chapel Hill; Carolina Population Center, 123 West Franklin St. Campus Box 8120, Chapel Hill, NC USA; 2 Public Health & Preventive Medicine, Oregon Health & Science University; 3181 SW Sam Jackson Park Road, Mail Code CB 669, Portland, OR 97239-3098, USA

**Keywords:** Epidemiology, United States, *Diet, Geographic Information Systems, Environment, Environment Design, Fast Foods, Restaurants, Young Adult, MeSH

## Abstract

**Background:**

Recent studies suggest that neighborhood fast food restaurant availability is related to greater obesity, yet few studies have investigated whether neighborhood fast food restaurant availability promotes fast food consumption. Our aim was to estimate the effect of neighborhood fast food availability on frequency of fast food consumption in a national sample of young adults, a population at high risk for obesity.

**Methods:**

We used national data from U.S. young adults enrolled in wave III (2001-02; ages 18-28) of the National Longitudinal Study of Adolescent Health (n = 13,150). Urbanicity-stratified multivariate negative binomial regression models were used to examine cross-sectional associations between neighborhood fast food availability and individual-level self-reported fast food consumption frequency, controlling for individual and neighborhood characteristics.

**Results:**

In adjusted analysis, fast food availability was not associated with weekly frequency of fast food consumption in non-urban or low- or high-density urban areas.

**Conclusions:**

Policies aiming to reduce neighborhood availability as a means to reduce fast food consumption among young adults may be unsuccessful. Consideration of fast food outlets near school or workplace locations, factors specific to more or less urban settings, and the role of individual lifestyle attitudes and preferences are needed in future research.

## Background

Neighborhood availability of fast food restaurants has recently received considerable attention as a target to prevent obesity [1-6]. It is intuitive that fast food restaurants contribute to obesity by promoting fast food consumption. However, few studies have tested the relationship between access to fast food and diet behavior and those that have rely on measures of fruit and vegetable intake [7-9]. Findings from an even smaller literature that investigates direct relationships with fast food consumption are mixed [10-12].

Furthermore, most evidence focuses on urban populations, with little research in suburban or rural populations. One of the difficulties is that urbanicity is often classified according to population density [13], which may correlate with cultural or social influences on diet and thus obscure important heterogeneity across urban, suburban and rural areas. Because the nature of accessibility in suburban or rural environments differ from urban environments, and other social, environmental, and individual influences of diet behavior may differ according to urbanicity, residential fast food restaurant availability may influence fast food consumption differently in rural, suburban, and urban contexts. Therefore, we hypothesize that the relationship between chain fast food availability with fast food consumption varies by urbanicity. In most existing research, generalizability across multiple contexts and comparisons by urbanicity are not possible due to small sample sizes and constrained geographic areas.

Additionally, variation in and limitations of neighborhood fast food availability measures may contribute to inconsistent and sometimes counterintuitive findings. Measures capturing fast food restaurants within a straight line (Euclidean) distance may obscure realistic proximity through usual routes of travel along roadways [6]. In contrast, network buffers, the polygon shaped by the street network up to a given distance, represent access to resources relative to the street network [14]. Whereas the count (or number) of fast food restaurants within a given area does not take into account population density and development, which are correlated with neighborhood food resource availability and independently related to dietary behavior [15]. Conceptually, greater density of food resources per capita may represent greater quantity and diversity of restaurant choices that may, in turn, influence decision for where and when to eat outside of the home. Fast food restaurant density per population [16,17] may therefore capture population density or serve as a proxy for physical development density. Alternatively, a roadway-scaled measure that represents the concentration of fast food outlets along access routes may help account for differences in fast food restaurant counts according to the amount of commercial activity.

We used national data from 13,150 sociodemographically diverse young adults living throughout the U.S to test the hypothesis that individuals living in neighborhoods with greater chain fast food availability report more frequent fast food consumption. We improve on prior fast food availability measures by examining fast food restaurants per roadway mile within 3 km street network distance from each respondent home, with sensitivity analysis comparing to measures used in published research. Capitalizing on the size and geographic scope of our study, we investigated how the association between fast food availability and consumption varies by urbanicity.

## Methods

### Study population and data sources

Our study sample is derived from respondents aged 18 to 28 years who participated in Wave III (2001-02) of the National Longitudinal Study of Adolescent Health (Add Health), a nationally representative, prospective cohort study of adolescents representative of the U.S. school-based population in grades 7 to 12 (11-22 years of age) in 1994-95 followed into adulthood. The Wave I Add Health study population (n = 20,745) was obtained through a systematic random sample of 80 high schools and 52 middle schools in the United States, stratified to ensure that the schools were representative of US schools with respect to region, urbanicity, school type, percentage of white students, and school size [18]. Respondents were followed through Wave II (n = 14,738, 1996) and Wave III (n = 15,197). Add Health included a core sample plus subsamples of selected minority and other groupings collected under protocols approved by the Institutional Review Board at the University of North Carolina at Chapel Hill. The survey design and sampling frame have been discussed elsewhere [18,19].

We used the Add Health Obesity and Neighborhood Environment database (ONEdata), a Geographic Information System that linked time-varying, community-level data to Add Health respondent Wave III home addresses geocoded with street-segment matches (n = 13,039), global positioning system (GPS) measurements (n = 1,204), and ZIP/ZIP+4/ZIP+2 centroid match (n = 685) among 14,322 Wave III respondents with sample weights. Residential locations were linked to attributes of areas within 1,3,5, and 8 km straight line distance (Euclidean neighborhood buffer) and through the street network (street network neighborhood buffer) surrounding each wave-specific respondent residence; and block group, tract, and county attributes from time-matched U.S. Census and other data, which were merged with individual-level Add Health interview responses [20]. The number of census block groups (n = 7,588) represents 3.6% of block groups included in the 2000 U.S. Census.

Of 14,322 Wave III respondents with sample weights, we excluded those reporting disability or pregnancy and Native Americans (due to sparse data) (n = 582). Remaining respondents missing individual and geographic data were also excluded (n = 590), leaving an analytic sample of 13,150. The analytic sample had higher parental income, had fewer children, were more likely to own a vehicle, and lived in more highly educated and lower poverty neighborhoods compared to those excluded for missing data.

### Study variables

#### Fast food intake

Weekly frequency of fast food consumption was ascertained from the question: "On how many of the past seven days did you eat food from a fast food place McDonalds, Kentucky Fried Chicken, Pizza Hut, Taco Bell, or a local fast food restaurant?"

#### GIS-derived neighborhood fast food availability data

Neighborhood fast food restaurant data were obtained from a commercial dataset of U.S. businesses corresponding to the Wave III interview period (2001). Fast food restaurants include a wide range of quick service establishments providing generally premade food and little table service; they include traditional burger outlets as well as delicatessens and coffee shops. To capture a more homogenous, well-defined category, we examined only chain fast food restaurants (e.g., McDonald's or Pizza Hut), classified according to the 8-digit Standard Industrial Classification (SIC) code 58120307 (fast-food restaurant, chain).

Kilometers of secondary/connecting and local, neighborhood and rural roads were obtained from StreetMap Pro (July 2003, v.5.2) data from Environmental Systems Research Institute (ESRI, http://www.esri.com) in Redlands, CA. We defined fast food availability as the number of chain fast food restaurants per 100 kilometer of roadway within a 3 km network buffer to account for differences in fast food restaurant counts according to the amount of commercial activity in an area. Our research with Add Health and a similar large population study suggests that restaurant proximity within a 3 km buffer is the most appropriate distance for examining associations between neighborhood fast food restaurant availability and individual level behavior [21,22].

#### Urbanicity

U.S. Census-defined urbanized areas (UA) were used to classify residential locations as non-urban (outside UA) or urban (inside UA). Urban locations were further distinguished as 1) low density [≤95% (75th percentile) developed land cover] and 2) high density [> 95% developed land cover] urban areas based on the area of developed land as a proportion of total area within 3 km after excluding water and ice (calculated using Fragstats software with U.S. Geologic Survey National Landcover Data). Our measure of developed land cover provides an indicator of urban development that is independent of population density, which may correlate with cultural or social influences on diet, and correctly classifies areas as within or outside of a UA (Receiver Operating Characteristic curve area = 0.937). Region was defined as Midwest, West, South, or Northeast.

#### GIS-derived neighborhood sociodemographics

We examined neighborhood sociodemographic characteristics within 2000 U.S. Census block groups because they more likely adhere to individually perceived neighborhood boundaries [23,24] and are more sociodemographically homogeneous than larger units. Using the federal definition of "poverty area" [25,26], we dichotomized neighborhood poverty into > 20% or ≤20% of population below the federal poverty level. We defined neighborhood-level education as percent of persons ≥25 years with a college degree. We obtained population density per squared kilometer by using Census block-group population count, weighted according to the proportion of block-group area within the 3 km neighborhood buffer, after excluding water and ice, and divided by the area.

### Individual characteristics

We adjusted for the following individual characteristics: race, age, parental income (> $36,000), employment status, has any children, vehicle ownership ("has a car/motorcycle/van"). We included parental rather than the young adults' own education (> high school) because parental SES is shown to be a strong predictor of obesity and obesity-related behaviors [27-29] during the complex transitional stage of young adulthood [30,31]

### Statistical analysis

All statistical analyses were carried out using Stata version 10.1 (Stata Corp, College Station, TX). Descriptive characteristics were calculated within each urban strata. In multivariable analysis, we used negative binomial regression to model individual-level, self-reported fast food consumption (days eating fast food in last week) as a function of neighborhood-level fast food restaurants per 100 kilometer roadway within 3 km network buffers, controlling for neighborhood sociodemographics, individual characteristics, and region. We hypothesized *a priori *that the association between fast food availability and fast food consumption is different across non-urban, low density urban, and high density urban areas. Because fast food outlets and neighborhood sociodemographics varied dramatically across urbanicity and thus limited comparability across sociodemographic and geographic subpopulations, we stratified by urbanicity to avoid structural confounding [32].

Our preference was to use consistent restaurant availability measures for all strata, but the alternative specifications yielded invalid estimates because they violated model assumptions and/or were highly unstable. For example, nearly 100% of the high density urban respondents had at least one fast food restaurant within 3 km of their home, so the dichotomous measure was unstable and relied on 60 observations. In contrast, approximately 70% of non-urban respondents had no fast food restaurants within 3 km, resulting in a highly skewed distribution of fast food availability and heteroskedasticity in our models. Thus, these distributions necessitated different measures across strata of urbanicity. We analyzed fast food availability as a continuous variable for low and high density urban strata and as a dichotomous measure (any versus none) for the non-urban stratum. Statistical interactions between fast food availability and vehicle ownership, poverty, and sex were not significant (p > 0.10) and therefore excluded from all models. We tested higher order relationships for all continuous variables and included where statistically significant (p < 0.05).

Parental selection of a neighborhood may be influenced by preferences or constraints related directly or indirectly to the area's food resources and diet behaviors, which may cluster within school populations. Thus, to address our concern that unobserved factors associated with fast food consumption might be associated with neighborhood choice and school at baseline (Wave I), we tested baseline school indicator variables in the models using a Hausman-like test [33], which indicated that the school indicator variables were necessary (p < 0.05). Thus, we include school indicator variables in our models to capture long lasting unobservable influences on individual decisions even after the individual leaves their place of residence at wave I.

Buffer-based fast food availability measures were individual-level variables. While block groups used for neighborhood-level control variables could comprise a third level in multilevel analysis, we did not perform multilevel modeling because census unit boundaries did not correspond with school catchment areas, so schools and census units were not hierarchically related. Therefore, analyzing census units as higher levels in multi-level models was not possible while controlling for school indicators, the primary sampling unit for Add Health. Furthermore, census block groups contained sparse, unbalanced numbers of respondents (mean 1.9, range 1-57 respondents), which can lead to bias in non-linear multi-level models, and clustering within census block groups was minimal (0.08 intraclass correlation for reported fast food consumption). We do, however, include school indicator variables in the models, which correct for school-level clustering and account for survey design.

### Sensitivity Analysis

We evaluated the sensitivity of our findings to the following aspects of our fast food availability measure (count of chain fast food restaurants per 100 kilometer roadway within 3 km street network buffers): (1) the variable used for scaling (roadway km vs. population), (2) network vs. Euclidean buffers, and (3) 1 km vs. 3 km buffers, and (4) total (chain and non-chain) vs. chain fast food restaurants. Corresponding alternative variables included (1) chain fast food restaurant counts per 10,000 population within 3 km Euclidean (straight-line) buffers (population counts were derived from 2000 US Census block-group population count weighted according to the proportion of block-group area within the neighborhood buffer); chain fast food restaurant counts per 100 kilometer roadway within (2) 3 km Euclidean buffers and (3) 1 km network buffers; and (4) total fast food (chain and non-chain) restaurant counts per 100 kilometer roadway within 3 km street network buffers.

We repeated our multivariable analysis using each of the four alternative fast food availability measures. Few non-urban and low density urban areas had any fast food restaurants within the 1 km network buffer (8% and 25%, respectively); therefore, we analyzed fast food availability within 1 km in high density urban areas only.

## Results

Our diverse national sample of 13,150 young adults reflects a variety of individual sociodemographic and neighborhood differences by urbanicity. In general, respondents of racial/ethnic minority lived in greater proportion in high density urban areas (Table 1). Joblessness and car ownership among respondents was highest in non-urban areas (Table 1). All neighborhood-level characteristics differed across urban strata (Table 2).

**Table 1:** Individual characteristics by urbanicity ^a^

	Non-urban	Low density urban	High density urban
	N = 3,662	N = 6,140	N = 3,348

Fast food consumption (days/week; mean)	2.5 (0.1)	2.4 (0.1)	2.5 (0.1)

Age (mean)	21.7 (0.2)	21.8 (0.1)	22.1 (0.2)

Male % (SD)	51.0 (1.1)	52.8 (1.1)	50.4 (1.4)

Race (%)			

White	78.7 (3.9)	70.8 (2.7)	45.8 (5.4)

Black	16.9 (3.7)	13.7 (2.0)	18.4 (3.2)

Asian	1.6 (1.2)	3.7 (0.6)	8.6 (2.4)

Hispanic	2.9 (0.6)	11.8 (1.5)	27.3 (4.8)

Household income > $36,000 (%)	47.4 (2.5)	59.5 (2.4)	45.1 (3.6)

Parental education > High School (%)	48.9 (2.1)	57.1 (1.9)	47.7 (3.4)

Has children (%)	32.9 (1.7)	26.6 (1.4)	31.1 (2.2)

Has job (%)	73.0 (1.5)	77.6 (1.2)	74.9 (1.5)

Owns vehicle (%)	78.7 (1.5)	74.8 (1.4)	66.8 (2.8)

^a ^National Longitudinal Study of Adolescent Health, Wave III (2001-2), n = 13,150. Weighted for national representation, standard errors corrected for survey design effects of multiple stage cluster sampling.

**Table 2:** Distribution of neighborhood characteristics^a^, National Longitudinal Study of Adolescent Health, Wave III (2001-2), n = 13,150

	Non-urban	Low density urban	High density urban
	**N = 3,662**	**N = 6,140**	**N = 3,348**

**Fast food availability measure**			

Chain fast food restaurant within 3 km network buffer around each individual residence			

Count per 100 km secondary and local road	0.6 (0.1)	1.4 (0.1)	2.2 (0.1)

Percent of respondents with count ≥1	27.8 (22.8,33.5)	79.0 (75.4,82.2)	98.6 (97.7,99.2)

*Alternate measures*			

Chain fast food restaurant within 3 km Euclidean buffer around each individual residence			

Count per 100 km secondary and local road	0.8 (0.1)	2.3 (0.1)	3.2 (0.2)

Count per 10,000 population	1.4 (0.1)	1.8 (0.1)	1.5 (0.1)

Percent of respondents with count ≥1	34.2 (28.1,40.8)	89.0 (86.5,91.1)	100.0 (99.9,100.0)

Chain fast food restaurant within 1 km network buffer around each individual residence			

Count per 100 km secondary and local road	0.5 (0.1)	1.3 (0.1)	1.9 (0.2)

Percent of respondents with count ≥1	8.7 (6.2,12.0)	24.6 (22.5,26.8)	44.6 (38.9,50.4)

Chain + non-chain ^d^ fast food restaurant within 3 km network buffer around each individual residence			

Count per 100 km secondary and local road	2.3 (0.2)	5.6 (0.2)	10.1 (1.1)

Percent of respondents with count ≥1	48.3 (42.6,54.0)	93.9 (92.3,95.2)	99.9 (99.2,100.0)

**Neighborhood demographics**			

Percent with college education or greater	16.8 (0.8)	25.8 (1.1)	22.6 (1.8)

Population density within 3 km Euclidean buffer around individual residence	155.6 (12.9)	1,206.2 (75.5)	3,612.2 (556.3)

Population > 20% are below 100FPL	31.2 (24.1,39.3)	20.9 (18.2,23.9)	36.4 (29.2,44.2)

Region			

West	7.8 (4.6,13.0)	18.6 (15.0,22.8)	12.9 (6.6,23.6)

Midwest	28.2 (19.7,38.6)	30.0 (23.6,37.3)	39.1 (27.8,51.6)

South	55.8 (46.4,64.8)	33.4 (28.1,39.2)	23.5 (14.1,36.6)

^a, b ^Means (SD)^c ^presented for continuous variables and percentage (95% CI)^c ^presented for categorical variables^c^.

^a ^Weighted for national representation, standard deviations corrected for survey design effects of multiple stage cluster sampling.

^b ^Non-urban: distance to Urbanized Area (UA) > 0, low density urban: distance to UA = 0 & % developed land cover, excluding water and ice (land developed) < = 95%, high density urban: distance to UA = 0 & % land developed > 95%.

^c ^Wald tests of means for continuous variables and Pearson Chi-Square tests for categorical variables were statistically significant (p < 0.005) across strata.

^d ^Chain and non chain outlets defined by SIC codes 58120307, 58120300-58120315, 58120600, 58120602.

Fast food availability was not associated with reported fast food consumption in non-urban, low density urban, or high density urban areas after controlling for individual and neighborhood characteristics (Table 3). In general, estimated associations were not sensitive to alternative definitions of fast food availability: the total fast food measure and chain fast food measures scaled by population and within different neighborhood buffers yielded similarly small and precise estimates. However, findings suggest that estimates may be sensitive to measure differences within the context of high density urban areas. A few estimates approached marginal significance but in inconsistent directions.

**Table 3:** Associations between fast food availability and reported days ate fast food, by urbanicity^a ^[beta coefficient (95% CI)]^b, c^, National Longitudinal Study of Adolescent Health Wave III (young adulthood; 2001-02)

	Non-urban	Low density urban	High density urban
	N = 3,662	N = 6,140	N = 3,348

Fast food availability measure			

Chain fast food restaurant within 3 km network buffer around each individual residence			

Count per 100 km secondary and local road	---	-0.01 (-0.03, 0.00)	0.02 (-0.01, 0.04)

Count ≥1 versus 0	-0.08 (-0.16, 0.01)	---	---

			

*Sensitivity analyses*			

Chain fast food restaurant within 3 km Euclidean buffer around each individual residence			

Count per 100 km secondary and local road	---	-0.01 (-0.02, 0.01)	0.02 (0.00, 0.04)

Ccount per 10,000 population	---	-0.01 (-0.02, 0.01)	0.05 (0.00, 0.09)

Count ≥1 versus 0	-0.03 (-0.11, 0.06)	---	---

Chain fast food restaurant within 1 km network buffer around each individual residence			

Count per 100 km secondary and local road	---	---	0.01 (0.00, 0.02)

Count ≥1 versus 0	---	---	---

Chain + non-chain ^e^ fast food restaurant within 3 km network buffer around each individual residence			

Count per 100 km secondary and local road	---	-0.01 (-0.01, 0.00)	0.00 (0.00, 0.00)

Count ≥1 versus 0	0.05 (-0.02, 0.13)	---	---

^a ^non-urban: distance to UA > 0, low density urban: distance to UA = 0 & % land developed ≤95%, high density urban: = distance to UA = 0 & % land developed > 95%

^b ^Negative binomial regression models, controlling for age, race, parental education, household income, owning car, relationship type, having any children, population density (except model of count per population measure), %college educated, employed (except where effect measure modification), > 20% population below FPL, % non-Hispanic White

^c ^Dashes represent un-estimated associations; 1) continuous measures in non-urban areas and dichotomous measures in low- and high-density areas 2) measure within network 1 km in non- and low-density urban areas.

^d ^Each sensitivity measure was modeled^b ^separately as continuous variables in low and high density strata and dichotomously in non-urban stratum.

^e ^Chain and non chain outlets defined by SIC codes 58120307, 58120300-58120315, 58120600, 58120602.

## Discussion

In contrast with our hypothesis, we found that neighborhood fast food availability was not related to fast food consumption in our large, national sample of young adults residing in neighborhoods throughout the U.S. Our findings suggest that targeting neighborhood fast food availability may not reduce consumption or obesity among young U.S. adults.

Our findings, of no relationship between fast food availability on fast food consumption among adults, are consistent with prior research [11,34]. One study reports a positive association between fast food availability and fast food consumption, but only among a subset of 404 adults living in Montreal who were "reward sensitive" [10].

Null results may reflect: (1) that young adults more often purchase and consume fast food in settings other than their residential neighborhoods, such as school or workplace locations, (2) that lifestyle factors such as family structure or employment status are stronger determinants of fast food consumption [35-37] or (3) the possibility that unmeasured neighborhood and social preferences, such as location selection factors, more strongly influence dietary behaviors. That is, the social, economic, cultural factors that affect where a person is able or wishes to live may also influence dietary behaviors [38]. Much would be gained by broadening research to focus on environmental contexts beyond the residential location, such as college and workplace neighborhoods as well as commuting routes. In addition, future research should incorporate other neighborhood- and individual-level factors to determine which settings and individual and neighborhood characteristics are most salient for dietary behaviors.

The majority of published research has focused on the indirect relationship between fast food availability and obesity, rather than investigating direct effects on fast food consumption. Greater neighborhood fast food availability is consistently related to higher obesity [1,3,39,40] yet it is possible that this relationship reflects processes other than higher fast food consumption. In particular, fast food restaurants may cluster with other environmental characteristics that influence obesity [20,41]. For example, automobile access may be important for fast food restaurants because of their dependence on drive-thru business. Therefore, neighborhoods with more fast food restaurants may promote obesity as a result of dominating road structures that hinder active transportation. Our findings that neighborhood fast food availability is unrelated to fast food consumption suggest that associations between fast food availability and obesity may reflect this or similar processes.

Our study expands current literature by comparing fast food restaurants scaled by roadway kilometers within a street network buffer - a measure of availability that improves upon widely used restaurant count measures by accounting for urban development and street access - across multiple geographic and sociodemographic characteristics. We present data from a large sample of U.S. young adults that uses a more refined urban/rural classification than the traditional urban/rural dichotomy. Furthermore, we observed vast differences in the availability of fast food for non-urban compared to urban respondents. While these differences precluded the use of comparable availability measures across urbanicity levels, they suggest that the built environment may operate quite differently in rural, suburban, and urban areas, and thus may necessitate different measurement approaches. This might also underlie some of the mixed findings in the literature [10-12].

### Strengths and limitations

We present data from a unique and large national cohort that includes a variety of detailed environmental data combined with individual-level diet data in a cohort of young adults from around the U.S. To our knowledge ours is the only large analysis of fast food availability and consumption that accounts for multiple environmental features and individual characteristics simultaneously.

Yet, our study has some limitations. Our measure of fast food consumption is based on self-report and, like all self-report diet measures, has inherent recall and reporting error. Respondents were not instructed how to report meals versus snacks; that is, snacking at a fast food restaurant may be undercounted if the respondent did not consider it as a visit. Further, because the question asks about the number of days that respondents ate fast food in the last week, fast food consumption may be under-represented if the respondent ate more than one fast food meal in a day. Yet, this is a measure that is commonly used to assess fast food consumption in large population-based studies such as the Panel Study of Income Dynamics and Coronary Artery Risk Development in Young Adults [39,42]. Moreover, we are unable to ascertain what foods were available and consumed at each fast food visit. This is increasingly an issue as fast food restaurants are including healthier options in response to consumer health concerns [43]. In addition, our study is cross-sectional and thus does not capture changes in the food environment or consumption over time. We were also unable to control for factors related to selection of residential neighborhoods, however by including Wave I school indicator variables, we attempted to address unmeasured characteristics associated with baseline neighborhood. Our 3 km network neighborhood buffer may not accurately reflect food-purchasing areas for different urban settings and sociodemographic subgroups, however estimates for the 1 km network buffer were very similar. Significant differences in characteristics of respondents included versus excluded in our sample may have biased our results.

There may be error in our roadway (StreetMap Pro) and food resource data that we are unable to investigate in our national sample. In addition, the relatively narrow range of fast food availability may limit our ability to detect effects of fast food availability in relation to fast food consumption. We were not able apply spatial interaction models to our national sample, but our models account for spatial clustering of respondents. The trade-off is our large, national sample and the ability to compare individuals living in non-urban, low density urban, and high density urban environments, within the context of the same cohort. There are no other datasets in which such a study is possible at the small, geographic unit used in our study.

Despite these limitations, our study is an essential step in understanding the allocation and consumption of fast food restaurants across geographic space over the entire U.S. and within urbanicity levels, and our findings can inform measurement and design in future individual-level and longitudinal studies.

### Implications

Our findings are significant in light of the recent efforts to reduce obesity though policies targeting the fast food environment. For example, a one-year ban on fast food restaurants was unanimously put forth in South Los Angeles (LA) in an effort to reduce obesity in this low SES area, despite lower fast food restaurant per capita relative to more affluent West LA [44]. Given evidence that eating fast food increases BMI and obesity risk [39,45], reducing fast food consumption may be a valuable aim. However, specific environmental factors that influence fast food consumption among young adults are not well understood. Our findings suggest that greater residential neighborhood fast food availability may not be an important driver of fast food consumption. Greater understanding of how lifestyle factors and neighborhood food resources interact to influence fast food consumption is needed to inform effective policy.

## Conclusions

Findings from our large, national sample of U.S. young adults do not support the hypothesis that neighborhood fast food availability increases the likelihood of fast food consumption among young adults. To understand the complex relationship between fast food availability and individual dietary behaviors, future research should investigate the settings in which young men and women consume fast food, other individual lifestyle and contextual influences on dietary choices, and how these processes differ across urbanicity and sociodemographic contexts.

## Competing interests

The authors declare that they have no competing interests.

## Authors' contributions

AR performed statistical analyses and drafted the manuscript. JBH and PGL guided statistical analyses and contributed to the manuscript. All authors participated in the study design, read and approved the final manuscript.

## Add Health data

The more extensive restricted-use data, available by contractual agreement, will be distributed only to certified researchers who commit themselves to maintaining limited access. To be eligible to enter into a contract, researchers must have an IRB-approved security plan for handling and storing sensitive data and sign a data-use contract agreeing to keep the data confidential.

## Pre-publication history

The pre-publication history for this paper can be accessed here:

http://www.biomedcentral.com/1471-2458/11/543/prepub
